# Evaluation of capabilities of Chinese provincial veterinary laboratories in detection of African swine fever virus: a proficiency testing program

**DOI:** 10.3389/fvets.2026.1785375

**Published:** 2026-03-30

**Authors:** Xiaoxue Gu, Zhenjie Zhang, Yingyi Liu, Xinxin Dong, Yang Liu, Yuan Zhang, Chuanbin Wang, Qi Xu, Xinyan Zhai, Ningwei Yu, Qi Li, Yuliang Liu

**Affiliations:** 1 China Animal Disease Control Center (CADC), Beijing, China; 2 College of Animal Science and Technology, Tarim University, Alar, China; 3 Zoetis Reference Laboratory, Shanghai, China

**Keywords:** African swine fever virus, proficiency testing, inter-laboratory comparison, Z-score, veterinary laboratory

## Abstract

**Introduction:**

Effective surveillance is essential for controlling African swine fever (ASF). Proficiency testing program (PTP) enhances the capability of veterinary laboratory in ASF surveillance and diagnosis.

**Methods:**

To evaluate and compare the capabilities of official provincial veterinary laboratories in China for detection of African swine fever virus (ASFV), a PTP based on inter-laboratory comparisons (ILCs) was implemented during 2020 and 2021. A six-item test panel was developed for the PTP using certified reference material (CRM), consisting of one negative control and five ASFV nucleic acid-positive samples at varying dilutions. A quantitative polymerase chain reaction (qPCR) was employed to detect the six coding samples. The qualitative evaluation of each participant was based on the consistency of the obtained results with the reference results. A Z-score evaluation system was utilized for quantitative assessment, which was further validated using a Youden plot and principal component analysis (PCA). Subsequently, the equipment, reagents and consumables used by the participants for testing were subjected to statistical evaluation.

**Results:**

Our results indicated that all laboratories (100%, 37/37) exhibited 100% consistency as expected. In the quantitative assessment, 89.19% (33/37) and 94.6% (35/37) of participants were qualified in the evaluations conducted in 2020 and 2021, respectively. Among the four participants who reported unsatisfactory results in 2020, one failed to detect two diluted weak positive samples, while the other three failed to detect strong positive samples. In 2021, two participants failed at both concentrations. There was no statistically significant difference in the method of nucleic acid extraction, reagents and consumables used in the PTP.

**Discussion:**

This study reports for the first time the systematic evaluation of the capacity of Chinese official veterinary laboratories in quantitative nucleic acid detection of ASFV via a two-year PTP, with qualitative detection serving as a basic competency verification. The results provide a reference for enhancing the capability of prevention and control of ASF in China.

## 1 Introduction

African swine fever (ASF) is a highly contagious disease caused by African swine fever virus (ASFV), resulting in significant mortality among both domestic and feral pigs of all ages (1). The outbreak of ASF has led to substantial economic losses for pig breeding farms and has considerably impacted the entire pig industry chain worldwide, owing to the disease’s high transmissibility and pathogenicity (2). Efficient, sensitive, and cost-effective methods for ASFV detection are crucial for establishing a reliable biosecurity barrier, which facilitates the identification and prompt interruption of infection sources and transmission routes (3). Nucleic acid testing (NAT) is the recommended methodology for ASFV detection with quantitative polymerase chain reaction (qPCR) that is being extensively implemented in laboratories of varying expertise and technical capabilities for the identification of ASFV (4).

The capacity of a veterinary laboratory to detect ASFV is a critical factor in assessing its suitability for ASFV detection (5). In China, the primary diagnostic platforms for detecting ASFV are located within veterinary laboratories across various institutions, including customs quarantine agencies, animal disease control centers, farms, slaughterhouses, and third-party testing laboratories, etc. It is standard practice for laboratories involved in ASFV detection to undergo capability assessments organized by regulatory authorities prior to or during projects, aiming at ensuring their testing capacity. Furthermore, these laboratories are required to comply with the standards established by the General Requirements for Laboratory Biosafety (GB 19489–2008) and the General Rules for Veterinary Laboratory Biosafety Requirements (NY/T 1948–2010).

The Proficiency Testing Program (PTP) is a crucial element of external quality assurance that employs Inter-Laboratory Comparisons (ILC) to assess the performance and accuracy of laboratory testing (6). As a national veterinary technical support organization, the China Animal Disease Control Center (CADC) is dedicated to implementing PTP for provincial animal disease control centers (ADCs) to enhance animal disease management, including ASF. In this study, we conducted two ILCs in 2020 and 2021 to evaluate the detection capabilities of provincial and municipal veterinary laboratories. Qualitative detection served as a fundamental competency filter, while quantitative detection constituted the core evaluation metric, providing a comprehensive reflection of the laboratories’ testing proficiency.

## 2 Materials and methods

### 2.1 Participating laboratories

A total of 37 laboratories participated in each ILC, encompassing all provincial and municipal ADCs in mainland China, which includes 32 provinces and autonomous regions, as well as 5 municipalities that are directly governed by the Central Government. Each participating provincial or municipal ADC institution was limited to a single independent group for the PTP. These laboratories were designated as core institutions for regional animal disease surveillance and detection, adhering to mandatory basic conditions: compliance with the General Requirements for the Competence of Testing and Calibration Laboratories (GB/T 27025), possession of qualified qPCR detection equipment for ASFV, and employment of professional technical personnel with certified ASFV detection skills. All 37 designated laboratories were officially invited to participate in the PTP, and all agreed to join and successfully completed the entire process of sample detection, result submission, and data feedback.

**Figure 1:**
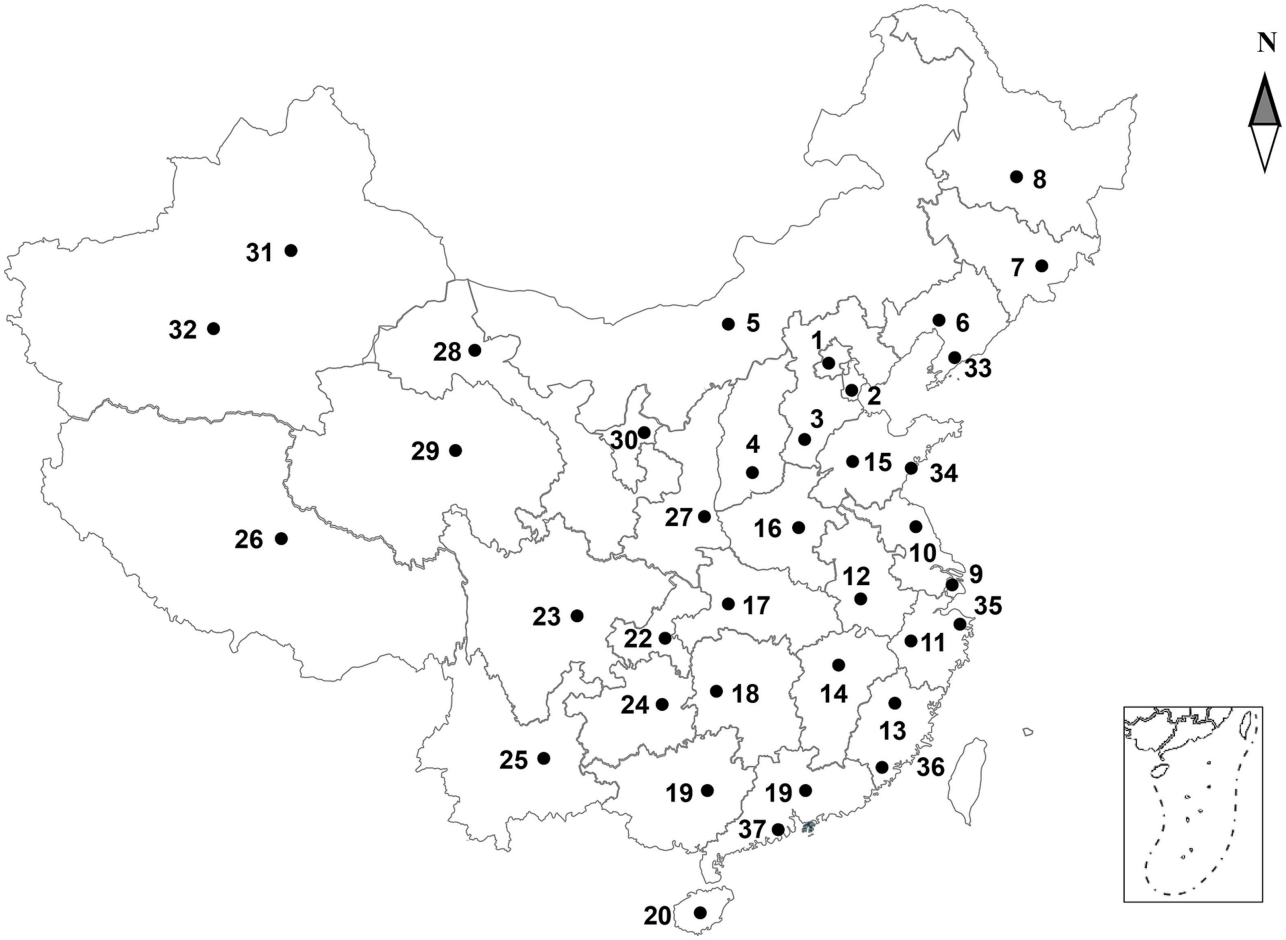
The geographical locations of the 37 laboratories involved in this study.

Each laboratory was assigned a unique and blind number during testing to ensure confidentiality (Figure 1). The unique blind numbers assigned to each laboratory are the only nomenclatures used for laboratory identification, result recording, and statistical analysis in this study, with no other alternative nomenclatures. All detection results and subsequent data analyses for each laboratory are uniquely corresponding to its assigned blind number.

### 2.2 Sample preparation and distribution

All samples for the ILC were prepared, aliquoted, labeled, and coded confidentially at CADC. The CRM for nucleic acid of ASFV was previously prepared by CADC, which was certified by National Standardization Administration of China with a certificate number of GBW(E) 091034. A panel of six samples was created, consisting of three weak positive samples with a concentration of 100 copies/μL of ASFV nucleic acid, two strong positive samples with 1,000 copies/μL of ASFV nucleic acid, and one sample containing phosphate-buffered saline (PBS) that served as a negative control. These samples were distributed to each participating laboratory for ASFV detection using qPCR.

To ensure blind testing, each sample in the package was assigned a confidential code composed of a six-digit number (e.g., 2,021,175), where the first four digits indicated the year and the last three digits denoted the sample number. A total of 37 packages, each containing six samples along with the results reporting form, were shipped via cold-chain transportation at −20 °C to the participating laboratories. This reporting form was formulated for the comprehensive collection of all required information to be submitted by each participant, including the confidential code of each blinded sample tested, the corresponding qPCR *Ct* values generated for each sample, as well as the detailed brand and supplier information of all instruments, equipment, reagents and consumables employed in the entire detection process (Supplementary Table S1).

### 2.3 Data acquisition

Each laboratory was authorized to utilize standard instruments and reagents for the detection of ASFV through qPCR. These instruments and reagents comply with the Chinese national standards and official technical specifications “Criterion on quality control of laboratories—Animal quarantine” (GB/T 27401) and have been officially approved for use in Chinese veterinary laboratories. Results were classified as “positive” (+) or “negative” (−), accompanied by their respective *Ct* values. Critical details regarding the methods and reagents used, as well as any modifications to the protocol, were documented as applicable. To ensure confidentiality, specific information pertaining to the testing conducted by each participating laboratory was withheld. All results were submitted to CADC via email by the respective participants, once the testing is completed.

### 2.4 Qualitative assessment

The qualitative detection assessment was based exclusively on the results reported by the participants in this study. These results were classified into three categories according to the viral loads of the blinded samples as described above, i.e., the group of 100 copies/μL ASFV group, the 1,000 copies/μL ASFV group, and the negative group. The participant’s performance in each group was deemed correct and satisfactory if their result, regardless negative or positive, was consistent with that of the reference; conversely, it was considered incorrect and unsatisfactory. It should be noted that, the results of one group did not affect the evaluation of any other groups, even within the same participating laboratory.

### 2.5 Quantitative assessment

The quantitative detection assessment was conducted based on the criteria that the *Ct* values, reference *Ct* values and converted evaluation values are all obtained. The term ‘reference values’ refers to the apparent errors or significant outliers identified and excluded during the initial stages of data analysis. These outliers often arose from variations in sample size for extraction and the volume of qPCR across the participating laboratories, leading to discrepancies in the actual copy numbers per tube. In this study, the coefficient of variation (CV) within groups was calculated, and a histogram was employed to evaluate whether the data obtained by the 37 laboratories were normally distributed.

The converted evaluation values were calculated as following: Z-scores were calculated using statistical analysis according to ISO/International Electrotechnical Commission (IEC) 17,043 and the International Harmonized Protocol (Equations 1, 2):


$$Z=\frac{\chi_{i}-\chi_{a}}{\sigma_{p}}$$ (1)

The value measured by participants was represented as the $X_{i}$ , the averaged value was denoted as the $\chi_{a}$ , and the standard deviation (SD) for ILC was indicated as $\sigma_{p}$ . Likewise, a Z-score between +2 and −2 was considered satisfactory; scores between +2 and +3 or between −2 and −3 were regarded as questionable, while any score falling outside the latter range was classified as unsatisfactory. Furthermore, if the degree of uncertainty of the assigned value (u, where u = $\chi_{a}/\sqrt[{2}]{n}$ ) is excessively high in comparison to the SD for ILC, the participants’ Z-scores may not accurately reflect their actual performance. Consequently, in this study, the u-values were specified (u ≤ $\sigma_{p}$ ) prior to calculating Z-scores in accordance with ISO 13528:2005 (E).

Classical parametric statistical methods were not employed in this study due to the skewed distribution and presence of outliers in the data of detection in 2021. These characteristics violate the normal distribution assumption necessary for parametric analysis, potentially resulting in biased outcomes. In contrast, robust statistical methods are resilient to outliers and non-normal distributions, thereby ensuring the accuracy and reliability of Z-score calculations. Accordingly, robust statistical methods are employed. The assigned value was calculated as the arithmetic mean of the participants’ results, considering the influence of outliers through robust statistical methods. The median value [med ( χ )] was selected to replace the average $\chi_{a}$ in order to more effectively mitigate deviation. Consequently, it was proposed that $\sigma_{p}$ be substituted with the normalized interquartile range (nIQR), which is based on robust statistical techniques. Specifically, nIQR was computed using the formula 0.7413 × (Quartile 3 - Quartile 1). Therefore, the aforementioned Z-score formula can be derived.


$$Z=\frac{\chi_{i}-\mathrm{med}(\chi )}{\text{nIQR}}$$ (2)

### 2.6 Youden plot analysis

To analyze the comparison blind sample data from the 37 veterinary laboratories, plot the Z-scores at 1000 copies/μL on the X-axis, and the Z-scores at 100 copies/μL on the Y-axis. Draw vertical and horizontal lines to delineate four quadrants. The points that are significantly distant from the data center in the figure are focused. If a laboratory does not adhere to the correct detection methodology, the results may be influenced by systematic errors, leading to substantial bias. Consequently, outlier points will be located in the lower left and upper right quadrants. Conversely, if random errors are present in the laboratory, outlier points will appear in the upper left and lower right quadrants.

### 2.7 Principal component analysis

In addition to Z-scores, Principal Component Analysis (PCA) was utilized to evaluate participants’ performance in this study. The raw *Ct* values from three replicates of 100 copies/μL ASFV nucleic acid samples and two replicates of 1,000 copies/μL ASFV nucleic acid samples, yielding five valid *Ct* values per laboratory, were selected for the PCA. The negative control sample, which lacked a valid *Ct* value, was excluded from the analysis. The PCA was conducted using GENALEX software, version 6.501 (http://biology-assets.anu.edu.au/GenAlEx).

## 3 Results

### 3.1 Qualitative evaluation of ILCs from different laboratories over two years

As anticipated, the results of the qualitative detection assessment during the two ILCs for ASFV detection in 2020 and 2021 were satisfactory for all participants (100%, 37/37) across the three sample groups, which were categorized based on the viral load of the blinded samples as described above (Table 1). In other words, all qualitative detection results were consistent with the reference values of the samples, with no instances of positive samples being incorrectly classified as negative, or vice versa. The ILCs were conducted independently in 2020 and 2021, and all results in this study were presented as separate data with detailed comparative analyses, rather than a simple summary of the data obtained in the two years (Table 2).

**Table 1:** Results of ILC for qualitative detection of ASFV.

Year of ILC	ILC results	Participating laboratory (n)			
		100 copies^a^ (3 replicates)	1,000 copies^b^ (2 replicates)	Negative samples	Satisfactory rate (overall)
2020	Correct	37	37	37	100%
	Wrong	0	0	0	
2021	Correct	37	37	37	100%
	Wrong	0	0	0	

^a^100 viral copies/μL of ASFV.

^b^1,000 viral copies/μL of ASFV.

**Table 2:** Summary of robust statistics analysis of ILCs.

Statistic parameters	Year 2020		Year 2021	
	100 copies^a^	1,000 copies^b^	100 copies^a^	1,000 copies^b^
Participant No.	37		37	
Minimum (*Ct*)	26.67	22.90	23.88	20.72
Maximum (*Ct*)	35.69	30.98	33.90	30.92
25% quantile (Q1)	30.24	25.99	29.64	26.45
75% quantile (Q3)	32.15	28.65	32.50	28.99
Median	31.27	27.30	31.18	27.59
MADe*	1.53	2.00	2.06	2.08
nIQR**	1.41	1.97	2.12	1.88
u*_MADe_*	0.31	0.41	0.42	0.43
0.3*σ_MADe_*	0.46	0.6	0.62	0.62
u*_nIQR_*	0.29	0.41	0.44	0.39
0.3*σ_nIQR_*	0.42	0.59	0.64	0.56

^a^100 viral copies/μL of ASFV.

^b^1000 viral copies/μL of ASFV.

*Median absolute deviation.

**Normalized inter quartile range.

**Figure 2:**
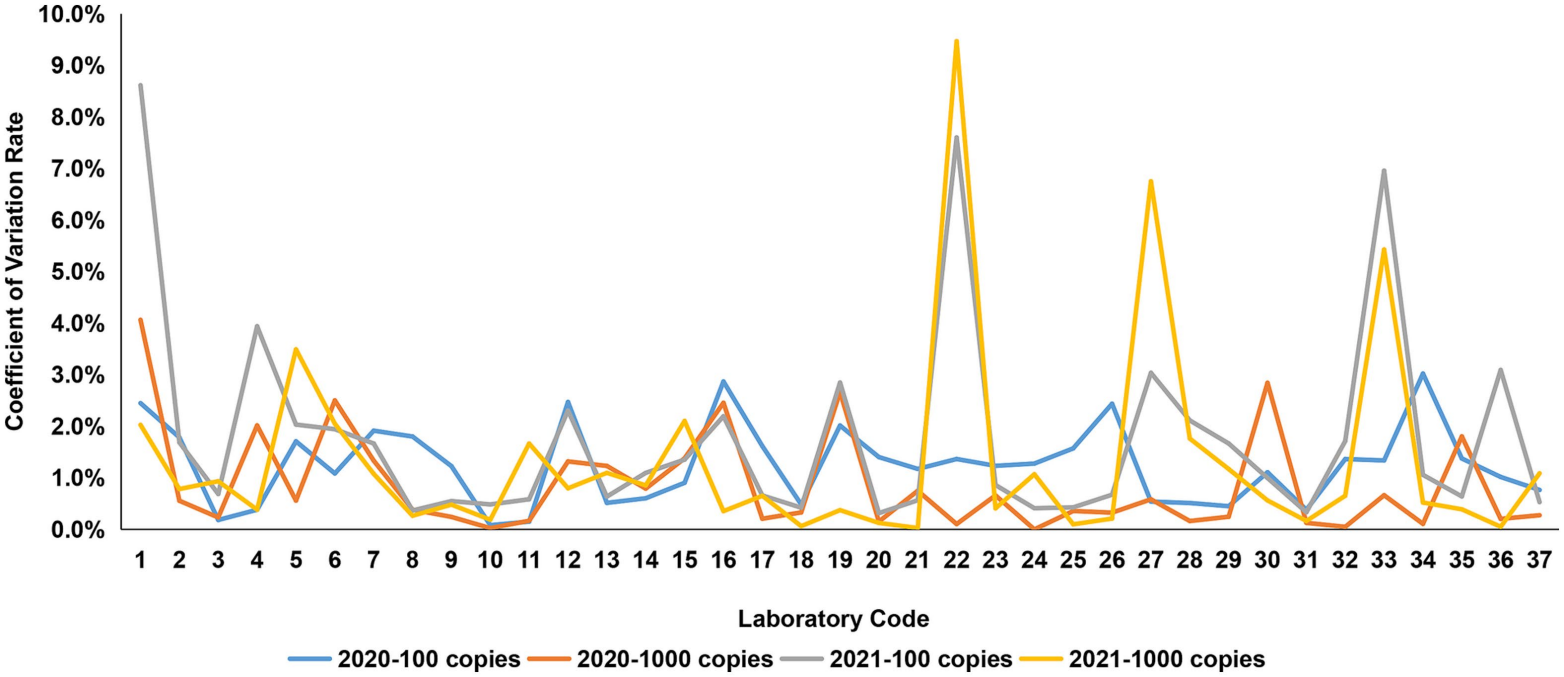
CV rate for African swine fever virus detection in the laboratory PT. The 37 participants were coded randomly, and the intra-CV values obtained from the results of the participants in the 2-year PTP were indicated.

**Figure 3:**
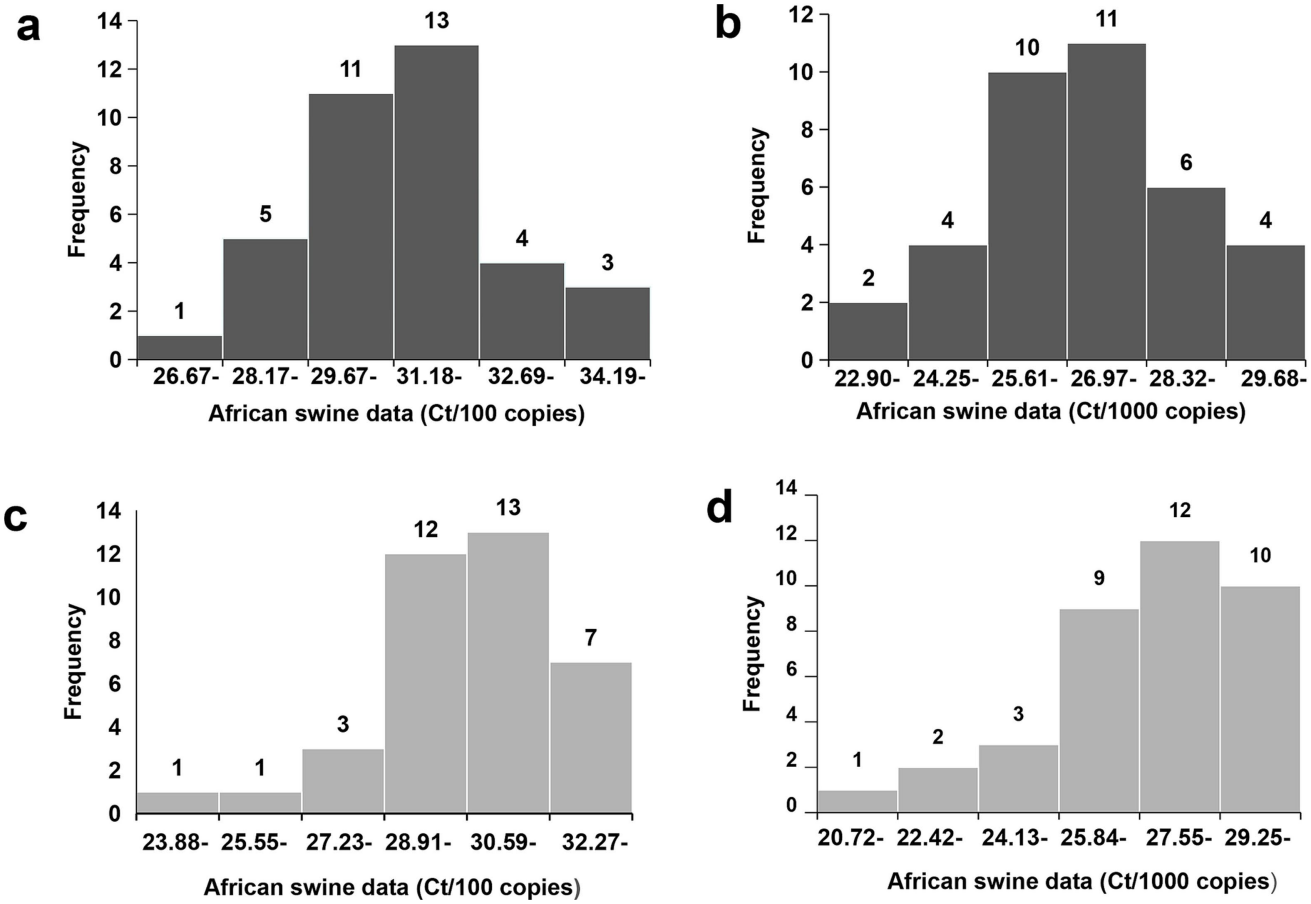
The histograms of raw data in the PT. The averaged *Ct* values obtained in 2020 **(a,b)** and 2021 **(c,d)** by each laboratory for samples with 100 copies/μL **(a,c)** and 100 copies/μL **(b,d)** were shown.

In the context of quantitative detection, one of the *Ct* values reported by participant #33 in the group with a viral load of 100 copies/μL in 2021 was 17.459, which was significantly lower than the value of that theoretically, as compared to the *Ct* values recorded by the same participant for the group with a viral load of 1,000 copies/μL. After official verification with the corresponding laboratory, it was known that this abnormal value was resulted from a technical operation error caused by sample cross-contamination during qPCR reaction setup, rather than a laboratory detection performance issue. To mitigate potential interference, these outliers were excluded from the analysis. The intra-CV values derived from the testing results of the 37 participants indicated that the range of intra-CV values for the two concentrations of tested samples in 2020 varied from 0 to 4%, demonstrating a generally smooth trend of change (Figure 2). In contrast, the highest intra-CV value, approximately 10%, was observed in 2021, indicating substantial variation among the obtained data (Figure 2). Furthermore, upon examining frequency histograms after calculating intra-averages of the testing results for each concentration sample, it became evident that the data from 2021 exhibited a skewed distribution when compared to that from 2020. Nevertheless, it is noteworthy that despite this difference, both years displayed a similar pattern, with each concentration of sample presenting a single peak (Figure 3).

The observed differences in CV values and the presence of skewed data distributions highlighted the necessity for robust statistical analysis to accurately assess detection capability. This analysis involved evaluating the quantile range from the 25th to the 75th percentile, the median values of each sample group over the two-year period, as well as the required interquartile range (IQR) values and u values through Z-score calculations. The results indicated that the u values (0.29, 0.41, 0.44, and 0.39) for each sample group were lower than the corresponding 0.3IQR values (0.42, 0.59, 0.64, and 0.56). Consequently, the findings demonstrated that the uncertainty associated with the median value for each group did not affect the accuracy of the Z-score calculations.

**Figure 4:**
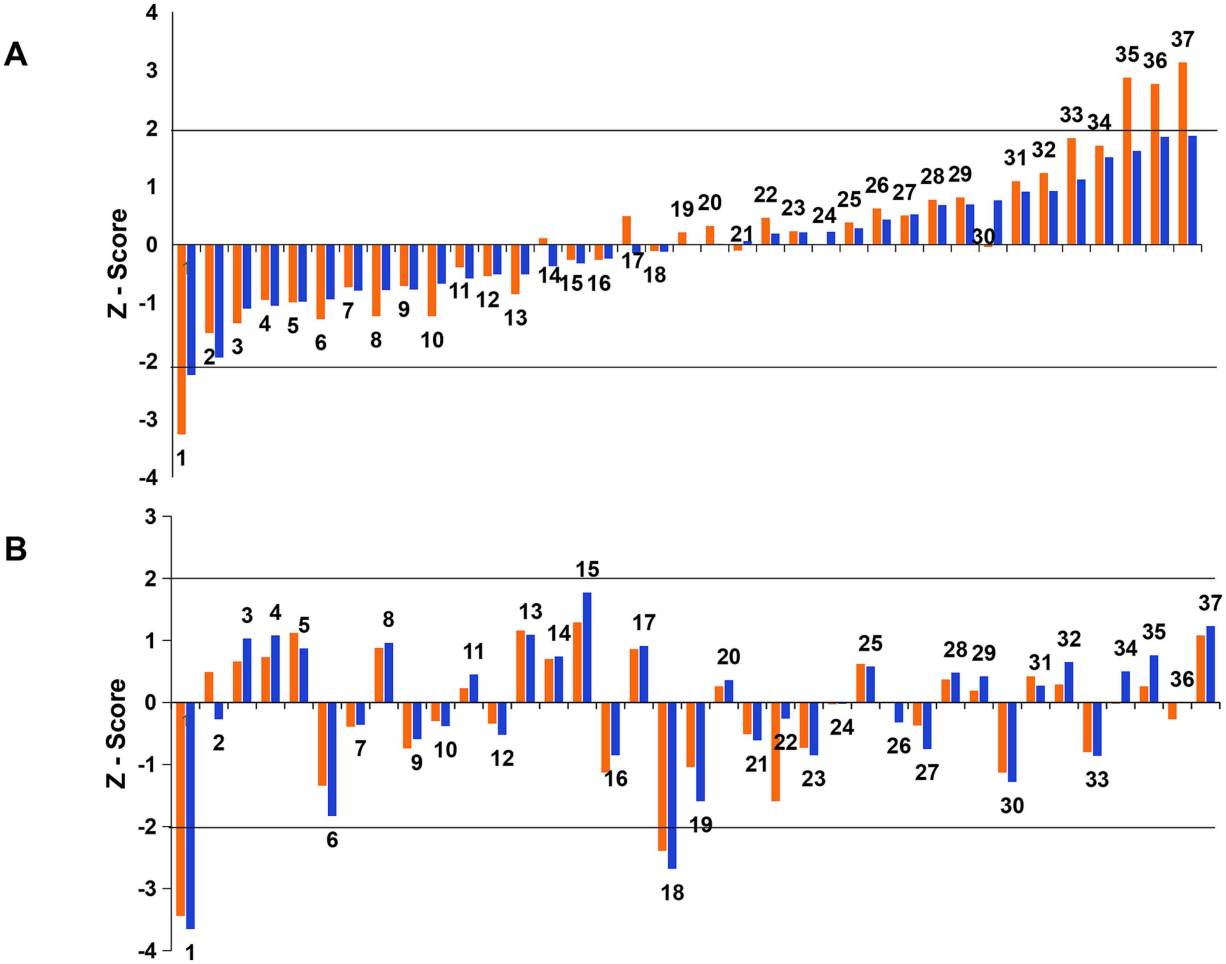
*Z*-scores for African swine fever virus detection in the laboratory PT in 2020 and 2021. The Z-scores of all 37 participants in the year 2020 **(A)** and 2021 **(B)** were calculated and shown in Y-axis, in which the blue and pink histograms represent samples with 1,000 and 100 copies/μL, respectively.

The Z-scores of all participants were calculated after adjusting for data and statistical methods. Among the 37 participants who submitted results in 2020 and 2021, 33 (89.19%) and 35 (94.6%) achieved satisfactory performance, respectively (Figure 4 and Table 3). In 2020, one participant was deemed unsatisfactory, failing in two groups (100 copies/μL and 1,000 copies/μL), while three others were unsatisfactory only in the group with a viral load of 100 copies/μL (Figure 4A and Table 3). Additionally, two unsatisfactory participants in 2021 failed in both concentrations mentioned above (Figure 4B and Table 3). The results of the ILC in 2020 indicated that most participants exhibited larger absolute Z-scores in the group with low viral concentration (100 copies/μL) compared to the group with higher viral concentration (1,000 copies/μL). Specifically, the Z-scores for both viral concentrations displayed a greater contrast in the four laboratories rated as unsatisfactory, three of which were considered unsatisfactory due to their failure to detect the low viral concentration, despite successfully detecting high viral concentrations that fell within the qualified range (Figure 4).

**Table 3:** Summary of satisfactory results in this study.

Statistic items	Year 2020		Year 2021	
	100 copies^a^	1,000 copies^b^	100 copies^a^	1,000 copies^b^
Assigned value (x*_a_*)	31.27	27.30	31.18	27.59
Standard deviation for proficiency assessment (*σ_p_*)	1.41	1.97	2.12	1.88
Satisfactory range (*Ct*)	28.45–34.09	23.36–31.24	26.94–35.42	23.83–31.35
No. of participants	37		37	
No. of satisfactory Participants	33	36	35	35
Satisfactory rate (Individual)	89.19%	97.30%	94.60%	94.60%
Satisfactory rate (overall)	89.19%		94.60%	

^a^100 viral copies/μL of ASFV.

^b^1000 viral copies/μL of ASFV.

### 3.2 Youden plot evaluation based on Z-scores

**Figure 5:**
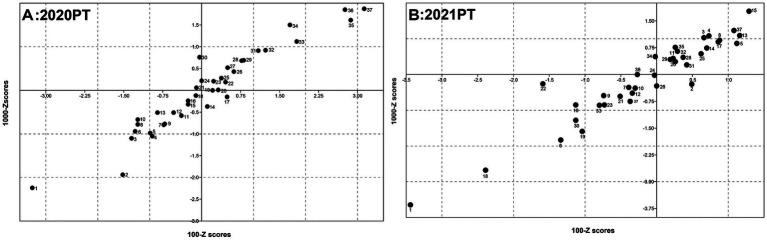
Z-scores of Youden plot for African swine fever virus detection in the laboratory PT. The Youden plot representing overall performance were generated based on the *Z*-score data of all participants in detection of samples with two viral concentration in 2020 **(A)** and 2021 **(B)**.

The Youden plot constructed from the Z-score data for the two viral concentration groups was employed to visually represent the overall performance of participating laboratories in the ILC. The performance of these participants displayed a greater degree of dispersion in 2020, compared to 2021. Notably, the results from 2020 indicated that the Z-scores of Laboratories #35, #36, and #37 were positioned in the upper right quadrant of the figure, consistently demonstrating a positive shift. In contrast, Laboratory #1 was situated in the lower left quadrant, exhibiting a negative shift (Figure 5). This observation may suggest the presence of systematic errors in these laboratories, leading to poor reproducibility. The results from the year of 2021 revealed that, excepting Laboratory #1 that demonstrated unsatisfactory results, all other laboratories that had large deviations in 2020 were classified into a reasonably coherent cluster (Figure 5).

### 3.3 PCA evaluation based on original *Ct* values

**Figure 6:**
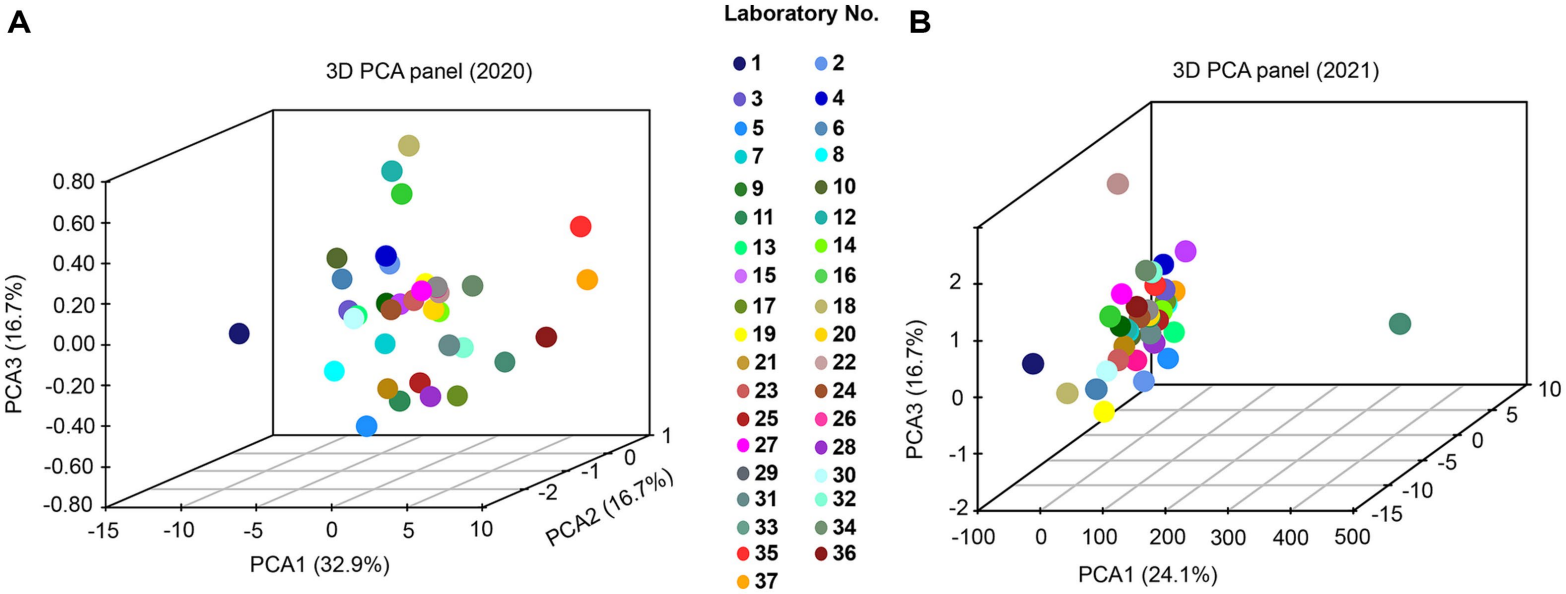
PCA for African swine fever virus detection in the laboratory PT. The PCA demonstrating degree of dispersion among the 37 participants were generated based on the original *Ct* values, as shown for the year of 2020 **(A)** and 2021 **(B)**, respectively.

The results of the PCA indicated a greater degree of dispersion among participants in 2020 compared to 2021, as illustrated in the 3D pattern plots (Figure 6). Notably, Laboratories #1, 12, 16, 18, 35, 36, and 37 were positioned at a distance from other clusters in 2020. In contrast, only Laboratories #1, 22, and 33 were identified as outliers in 2021 (Figure 6).

### 3.4 Evaluation of testing reagents and equipment used in 2021 ILC

Based on the results reported by 37 participants in 2021, and utilizing Z-score statistics, we evaluated the satisfaction levels related to the nucleic acid extraction methods, reagents, and instruments used in this proficiency testing (PT). The findings indicated no statistically significant difference in satisfaction between the Magnetic beads method (94.12%, 32/34) and the column extraction method (100%, 3/3) (*χ^2^* = 0.187, *p* = 0.67, 95%CI: 0.865–1.024). Furthermore, the satisfaction rates for domestic and imported nucleic acid extraction reagents were 93.75% (30/32) and 100% (5/5), respectively (*χ^2^* = 0.33, *p* = 0.57, 95%CI: 0.857–1.025). In terms of nucleic acid extraction equipment, the satisfaction rates were 89.17% (17/19) for domestic and 100% (18/18) for imported equipment (*χ^2^* = 2.003, *p* = 0.157, 95%CI: 0.767–1.044). These results suggested that imported reagents and equipment performed better than their domestic counterparts, however, no statistical differences were observed in the comparisons. Additionally, all PCR amplification reagents utilized by the 37 laboratories were sourced domestically, while all fluorescence quantitative PCR instruments were imported, yielding a combined satisfaction rate of 94.59% (35/37) for both categories. The aforementioned statistical results are concurrently presented in Table 4.

**Table 4:** Summary of testing reagents and equipment used in 2021 ILC based on *Z*-score.

Method	No. of participants using the method	No. of participants with satisfactory results	Satisfactory performance rate, %	*χ^2^, P, CI*
Nucleic acid extraction method				
Magnetic beads	34	32	94.12	0.187, 0.67, 0.865–1.024
Column	3	3	100	
Nucleic acid extraction reagent				
Domestic	32	30	93.75	0.33, 0.57, 0.857–1.025
Imported	5	5	100	
Nucleic acid extraction instrument				
Domestic	19	17	89.47	2.003, 0.157, 0.767–1.044
Imported	18	18	100	
PCR amplification reagent				
Domestic	37	35	94.59	/
Imported	0	0	0	
Fluorescence quantitative PCR instrument				
Domestic	0	0	0	/
Imported	37	35	94.59	

## 4 Discussion

The detection of ASF in laboratory settings plays a crucial role in the prevention and control of the disease. Recommended methods for ASFV detection, such as PCR and qPCR, are widely utilized (7, 8). The accuracy of laboratory assays is directly influenced by various factors related to detection, including operators, instruments, materials, methods, and environmental conditions (9, 10). Although ILCs for the qualitative detection of ASFV have been conducted, quantitative detection has been lacking, as organizers and participants often prefer simpler and more reliable evaluation parameters to meet minimum testing qualification requirements. Furthermore, the analysis of ILCs for quantitative detection of ASFV necessitates complex statistical methods, which may challenge organizers in establishing evaluation criteria and drawing clear conclusions. This study, organized by CADC, addressed these limitations by utilizing CRM for the nucleic acid of ASFV to evaluate the capacity of different veterinary laboratories through quantitative analysis in compliance with Z-score evaluation criteria, combined with Youden plot and PCA validation. To the best of our knowledge, this is the first report describing national ILC activity for ASFV quantitative detection in mainland China.

In this study, the qualitative testing results indicated that all participants met the judgment requirements, while the quantitative testing results exhibited slight variability. Among the 37 laboratories that submitted results in 2020 and 2021, a total of 33 (89.19%) and 35 (94.6%) performed satisfactorily, respectively. The number of unsatisfactory participants did not differ significantly between the two consecutive years. According to ISO/IEC 17043, approximately 10% of Z-values are expected to fall outside the qualified range if statistical methods and data are error-free (11). The occurrence of unqualified signals in large numbers is unlikely unless there is a discernible cause for the abnormality (12). To ensure the absence of error caused by experimental manipulations, in this study outliers were eliminated from the original data using robust statistical methods as previously reported (13). The results presented in this study revealed that, although most participants maintained qualified capabilities for ASFV detection over the two years, some participants reported results with systematic errors.

The data requirements for determining Z-scores are inherent, and *Ct* values, commonly utilized in pathogen detection, may not be suitable for such methods, despite their widespread application in ILCs for viruses such as SARS-CoV-2 and the rabies virus (14, 15). *Ct* values, derived from the conversion of fluorescence signals to quantify nucleic acid load, somewhat compromise the integrity of the data (16). The calculation of Z-scores involves various formulas, including the estimation of the median and the nIQR. It is not too surprising that the inclusion of additional estimated measures may further distort raw data from their true values. In this study, we aimed to employ preliminary PCA-based dimensionality reduction calculations on raw *Ct* values to avoid estimator operations that could jeopardize data accuracy (17). This approach enabled the direct identification of clusters and outliers among the participating laboratories, although this strategy is not currently addressed by ISO/IEC 17043 regulations or other inter-laboratory PT. Nevertheless, it supports the validation of results from quantitative PT projects that utilize *Ct* values as outcomes as previously demonstrated (18, 19).

In this study, PT was evaluated for provincial and municipal Animal Disease Control (ADC) laboratories in China. These 37 laboratories were selected due to their comparatively higher detection capabilities, which can be attributed to superior technical operators and operating environments in comparison of other veterinary laboratories, such as those located in counties or farms. As previously mentioned, ASFV detection is currently performed in various types of laboratories across China; thus, it is essential for these facilities to participate in PT programs, such as ILC described herein to enhance their understanding of their capabilities. The findings presented in this study may serve as a reference for governmental authorities and agencies in the context of ASFV detection and surveillance. However, it is important to note that, the limited number of PT programs discussed in this study may not fully reflect the entire competencies or skill levels of the participating laboratories of the whole China, as anomalous data can occasionally arise even in well-functioning laboratories. Furthermore, some methods and adjudication principles outlined in standards such as ISO/IEC 17025 and ISO 13528, may not be entirely consistent when applied across different scenarios for varying project requirements, an inconsistency that may only become apparent after multiple rounds of PT (20). Therefore, this study acknowledges certain limitations, including the restricted number of PT programs conducted and the types of laboratories involved. To identify deficiencies among participants, multiple PTs should be conducted regularly, followed by timely corrective actions to enhance the detection capabilities of the laboratories (21, 22). Our follow-up communication indicated that the outlier from Participant #33 in 2021 was a technical operation error rather than an inherent detection capability defect, which further indicates that standardized experimental operation and strict quality control are important guarantees for the accuracy of qPCR detection results, in addition to the laboratory’s hardware equipment and reagent conditions.

Numerous factors influence the results of qPCR, encompassing both unquantifiable and statistical elements, such as operator proficiency, equipment, and the testing environment. In the ILCs of this study, the absence of restrictions on the sources of commercial kits used by participants for nucleic acid extraction and/or amplification may complicate the determination of whether differences in detection capabilities among laboratories are attributable to specific kits or instruments. Nonetheless, the data and analysis presented in this study indicated no statistical difference between domestic and imported kits and instruments. This finding suggests that domestic reagents and/or instruments may be effectively utilized in laboratories for routine quantitative monitoring of ASFV and/or for emergency surveillance during an outbreak, thereby mitigating the time and cost associated with procuring foreign products. However, further experiments are necessary to substantiate and validate this conclusion.

## 5 Conclusion

This study reports the first ILC for ASFV detection in mainland China. The results, evaluated using Z-scores, indicate that majority of provincial and municipal laboratories in mainland China have demonstrated their qualification. These findings not only provide a theoretical basis but also serve as a valuable reference for ASFV detection in laboratory.

## Data Availability

The raw data supporting the conclusions of this article will be made available by the authors, without undue reservation.
