# Supplementary material for: Evaluation of capabilities of Chinese provincial veterinary laboratories in detection of African swine fever virus: a proficiency testing program
Source: Front Vet Sci. 2026 Mar 30;13:1785375. doi: 10.3389/fvets.2026.1785375 (PMC13070768; doi:10.3389/fvets.2026.1785375)
Supplement: Supplementary file 1 [file Table_1.DOC]

**Table S1 Statistical table of African Swine Fever Virus nucleic acid testing results**

| Sample original number |  |  |  |  |  |  |
| --- | --- | --- | --- | --- | --- | --- |
| Test result |  |  |  |  |  |  |
| *Ct* value |  |  |  |  |  |  |

Note: Fill in “Negative (-)” or “Positive (+)” for the test result, and keep the *Ct* value to two decimal places. If the *Ct* value is not detected, write “None”. The brand and name of the extraction and amplification reagents used for the test are:
